# The *IL17F* His161Arg polymorphism, a potential risk locus for psoriasis, increases serum levels of interleukin-17F in an Asian population

**DOI:** 10.1038/s41598-019-55062-5

**Published:** 2019-12-12

**Authors:** Byung Gon Choi, Ji Youn Hong, Joo Ran Hong, Min Seok Hur, Sung Min Kim, Yang Won Lee, Yong Beom Choe, Kyu Joong Ahn

**Affiliations:** 1 Department of Dermatoogy, Konkuk University School of Medicine, Seoul, Korea; 2 Research Institute of Medical Science, Konkuk University School of medicine, Seoul, Korea

**Keywords:** Skin diseases, Risk factors

## Abstract

Interleukin 17 (IL-17) plays pivotal role in the pathogenesis of psoriasis. In a previous study, we identified a locus in the *IL17F* gene that is associated with psoriasis, the *IL17F* rs763780 (His161Arg) T/C variant. The current study aimed to elucidate the association between this polymorphism and psoriasis, and to determine its effect on serum levels of cytokine. A total of 116 psoriasis patients and 97 healthy volunteers were recruited. Genotyping analysis was performed using quantitative polymerase chain reaction, and serum levels of cytokine were measured using a multiplex immunoassay. The *IL17F* His161Arg polymorphism was significantly associated with psoriasis based on the genotype and allele analyses. Psoriasis patients harbouring the mutant allele had significantly increased serum levels of IL-17F. Our results suggest that this polymorphism is a potential risk locus for psoriasis and that it results in a direct increase in IL-17F production.

## Introduction

Psoriasis is a representative, systemic inflammatory dermatological disease, for which specific immunological mechanisms have been reported^1–3^. Based on these investigations, the therapeutic paradigm of psoriasis has been expanded to include biological agents that target specific molecules associated with immunopathogenesis, as well as systemic immunomodulatory agents, such as methotrexate and cyclosporine^1,4^.

Genetic predisposition is also an important risk factor for psoriasis. Population studies have revealed some genetic factors associated with psoriasis, including ethnicity and common hereditary background (for example, between twins and first- or second-degree relatives)^2,5^. From genetic studies, various genetic loci have been significantly associated with psoriasis^5,6^. We previously conducted a preliminary study to investigate loci that have been identified in other inflammatory diseases but have not yet studied in psoriasis. Our study revealed the rs763780 (7488 T/C) psoriasis susceptibility single nucleotide polymorphism (SNP) in the *IL17F* gene on chromosome 6, which encodes interleukin-17F (IL-17F)^7^. This is a missense mutation that results in a histidine-to-arginine substitution at amino acid 161 (His161Arg).

IL-17F is a member of the IL-17 family of cytokines and is important in inflammatory and autoimmune diseases^8,9^. A previous study on the *IL17F* His161Arg variant in patients with asthma showed that this polymorphism is protective against asthma and characterised the function of this mutant at the molecular level^10^. In psoriasis, IL-17A secreted from type 17 helper T cells has a pivotal role in pathogenesis. IL-17A induced inflammation has been associated with an increased risk of cardiovascular diseases^11^. Among the IL-17 family, IL-17F is most similar with IL-17A and mediates the signaling pathway through the same receptors as IL-17A^12,13^. Furthermore, the levels of IL-17A and IL-17F are increased in the lesional skin and blood of patients with psoriasis^14^. Based on these investigations, biological agents targeting the IL-17 pathway, including secukinumab and ixekizumab, have been approved, and have contributed to the breakthrough in the treatment of psoriasis^15,16^. Moreover, agents targeting both IL-17A and IL-17F, such as ALX-0761 and bimekizumab, are currently in clinical trials^17^.

Here, we aimed to study the association between the *IL17F* His161Arg polymorphism and the clinical manifestations of psoriasis and to determine its effect on serum levels of cytokine. Since the results of our preliminary study had not been corrected for multiple comparisons, we also performed genotype analysis using normal controls to confirm that this is a susceptibility locus. In addition, cytokine analysis was performed in the control group to determine whether this polymorphism affects cytokine levels in the general population. To the best of our knowledge, this is one of only a few studies conducted on the effect of the *IL17F* His161Arg polymorphism on psoriasis, and is only the second study (after our previous study) of its kind in an Asian patients. Our study reveals an association between the *IL17F* His161Arg variant and psoriasis risk, and its effects on serum levels of cytokines in this population.

## Methods

### Study subjects

Patients and healthy volunteers who visited the dermatology clinic at Konkuk University Hospital from February 2016 to May 2017 were recruited. Psoriasis was diagnosed based on clinical and histopathological examinations by expert dermatologists. Subjects with a history of asthma, Crohn’s disease or ulcerative colitis, all of which have been reported to be associated with the *IL17F* rs763780 polymorphism, were excluded^18–20^. Subjects with autoimmune or autoinflammatory diseases, such as rheumatoid arthritis and systemic lupus erythematosus, which can affect serum inflammatory cytokine levels, were also excluded. Among the controls, subjects with a history of psoriasis, palmoplantar pustulosis, psoriatic arthritis or other papulosquamous diseases that can mimic psoriasis were excluded. Data, including age of onset, psoriasis area and severity index (PASI) score, disease duration, history of psoriatic arthritis, family history of psoriasis and specific phenotypes such as large plaque psoriasis or guttate psoriasis, were documented.

This study was conducted according to the Declaration of Helsinki and Korean Good Clinical Practice, and was approved by the institutional review board of Konkuk University Hospital, Seoul, Korea (approval number: KUH1120089). Written informed consent was obtained from all study participants.

### Genotyping analysis

Peripheral blood samples were collected from all psoriasis patients and control subjects. Blood samples of 3–5 ml were collected in two separate vacuum tubes: ethylenediamine-tetra acid (EDTA) tubes for genomic DNA extraction and serum separating tubes for serum preparation. The prepared samples were then stored at −70 °C until analysis.

The participants were genotyped for the *IL17F* T/C (rs763780) polymorphism. Genomic DNA was isolated from the blood collected in EDTA tubes using the Exgene™ Blood SV mini kit (GeneAll Biotechnology, Seoul, Korea) and genotyped using predesigned TaqMan® SNP genotyping assays (assay identification number C___2234166_10; Applied Biosystems, Foster City, CA, USA). All procedures were performed according to the manufacturer’s instructions.

### Serum cytokine analysis

Fourteen inflammation-related cytokines reported to play a significant role in the development of psoriasis were analysed: IL-1 receptor antagonist (IL-1RA), IL-2, IL-6, IL-8, IL-10, IL-12 (p70), IL-17A, IL-17F, IL-21, IL-22, IL-23 subunit alpha (IL-23A), tumor necrosis factor alpha (TNF-α), interferon alpha (IFN-α), and IFN-γ^2,3,5^. Since IL-12 subunit beta (p40) is shared with IL-23, the active heterodimeric form of IL-12 (p70) and the subunit alpha (p19) of IL-23 were targeted for analysis.

To analyse levels of all cytokines simultaneously, a multiplex immunoassay with customised kits (ProcartaPlex™ Luminex bead-based multiplexing assay; eBioscience, Vienna, Austria) was used. All procedures were performed according to the manufacturer’s instructions. Briefly, each sample, bead solution, bead diluents, and buffer were placed into the standard well. After beads were added into the mixtures, the plate was incubated at 4 °C overnight. Detection antibodies and streptavidin-phycoerythrin (PE) were added in order at room temperature. The plate was analysed, a standard curve was obtained and concentrations of the samples were measured by the fluorescence intensity.

### Statistical analyses

Student’s *t*-test and Mann-Whitney test were used for the comparison of continuous data. Chi-square tests and Fisher’s exact tests were used to compare categorical data between two groups. Continuous data are expressed as the mean and standard deviation (SD) or median and interquartile range (IQR) as representative values for the *t*-test and Mann-Whitney test, respectively. The observed genotype distribution of the target SNP was tested for deviation from Hardy–Weinberg equilibrium using the chi-square test. For genotype and allele analysis, logistic regression analysis was used, and adjusted odds ratios (ORs) with 95% confidence intervals (CIs) were obtained. SPSS software version 24.0 (SPSS Inc., Chicago, IL, USA) was used for statistical analyses. *P*-values < 0.05 were considered statistically significant.

## Results

### Characteristics of study subjects

A total of 116 patients with psoriasis and 97 healthy volunteers were recruited. The age range was broader in the psoriasis group (13–68 years) than in the control group (22–65 years), and the mean (SD) age was significantly higher in the psoriasis group [39.5 (14.8) years] than in the control group [35.2 (11.0) years, *P* = 0.019]. There was no significant difference in sex distribution between the two groups (*P* = 0.328, Table 1). Clinical information regarding psoriasis, including age of onset, PASI score, disease duration, history of psoriatic arthritis and family history of psoriasis are described in Table 1.

**Table 1:** Demographic data for control and psoriasis subjects.

	Control subjects	Psoriasis subjects	*P*-value
Total number	97	116	
**Age**, **years**			
Mean (SD)	35.2 (11.0)	39.5 (14.8)	**0**.**019**
**Male sex**			
Number (%)	47 (48.5)	64 (55.2)	0.328
**Onset age**, **years**			
Mean (SD)		28.6 (14.9)	
**PASI score**			
Mean (SD)		9.3 (5.4)	
**Duration**, **years**			
Median (IQR)		9 (3–15)	
**Psoriatic arthritis**			
Number (%)		5 (4.3)	
**Family history**			
Number (%)		30 (25.9)	

Abbreviations: IQR, interquartile range; PASI, psoriasis area severity index; SD, standard deviation.

### Genotype and allele frequencies

Both the psoriasis and control groups complied with Hardy–Weinberg equilibrium (*P* = 0.786 and *P* = 0.516, respectively). Table 2 shows the genotype and allele frequencies for control and psoriasis subjects. The minor allele frequency was 0.12 for the psoriasis subjects and 0.06 for the control subjects. The minor allele, C, was significantly associated with an increased risk of psoriasis compared to that with the major allele, T (OR = 2.36, 95% CI: 1.15–4.85; *P* = 0.019). Overall, homozygous CC genotypes with two minor alleles were observed in the psoriasis group, but not in the control group. Based on the dominant model, TC or CC genotypes appeared to be significantly associated with an increased risk of psoriasis compared to that with the TT genotype (OR = 2.28, 95% CI: 1.05–4.92; *P* = 0.037). Analyses based on the recessive model and CC genotype versus TT genotype showed no statistical significance because there was no CC genotype in the control group.

**Table 2:** Genotype and allele frequencies of the *IL17F* rs763780 polymorphism in control and psoriasis subjects.

*IL17F* rs763780	Controls (n = 97)	Psoriasis (n = 116)	OR (95% CI)*	*P*-value
**Genotype**				
TT	85	90	Reference	
TC	12	24	1.98 (0.92–4.27)	0.080
CC	0	2	—	0.999
**Dominant**				
TT	85	90	Reference	
TC + CC	12	26	2.28 (1.05–4.92)	**0**.**037**
**Recessive**				
TT + TC	97	114	Reference	
CC	0	2	—	0.999
**Allele**				
T	182	204	Reference	
C	12	28	2.36 (1.15–4.85)	**0**.**019**

Abbreviations: CI, confidence interval; OR, odds ratio

*Odds ratios adjusted by age and sex.

### Serum levels of cytokine

Cytokine analyses were performed to investigate the effect of the mutant allele of the *IL17F* rs763780 polymorphism on serum levels of cytokine in the control and psoriasis groups. For comparison, each group was divided into TT and TC + CC groups (Table 3). In the control group, there was no significant difference in serum levels between the TT and TC + CC groups for any of the 14 cytokines. However, in the psoriasis group, serum levels of IL-17F and IL-12 were significantly higher in the TC + CC group than in the TT group (*P* = 0.049 for IL-17F and *P* = 0.027 for IL-12; detailed information provided in Table 3). As IL-17F was undetectable in many subjects, serum levels are expressed as the median (range) rather than median (IQR).

**Table 3:** Differences in serum levels of cytokine according to *IL17F* rs763780 genotypes in control and psoriasis subjects.

*IL17F* rs763780	Control subjects			Psoriasis subjects		
	TT group	TC + CC group	*P* -value	TT group	TC + CC group	*P* - value
(pg/ml)	Median (Q1–Q3)	Median (Q1–Q3)		Median (Q1–Q3)	Median (Q1–Q3)	
IFN-α	—	—		0.00 (0.00–0.00)	0.00 (0.00–0.51)	0.115
IFN-γ	0.00 (0.00–4.06)	0.00 (0.00–6.31)	0.536	0.00 (0.00–4.23)	0.00 (0.00–7.37)	0.814
IL-1RA	—	—		0.00 (0.00–419.22)	0.00 (0.00–252.78)	0.890
IL-2	—	—		0.00 (0.00–9.79)	0.00 (0.00–11.42)	0.407
IL-6	—	—		0.00 (0.00–6.41)	0.00 (0.00–4.23)	0.861
IL-8	—	—		4.78 (0.00–9.02)	3.02 (0.00–10.70)	0.883
IL-10	0.00 (0.00–0.00)	0.00 (0.00–1.48)	0.370	0.00 (0.00–0.21)	0.00 (0.00–0.29)	0.968
**IL-12 (p70)**	0.00 (0.00–0.00)	0.00 (0.00–1.11)	0.936	**0**.**00 (0**.**00**–**3**.**01)**	**1**.**77 (0**.**00**–**6**.**80)**	**0**.**027**
IL-17A	0.00 (0.00–1.20)	0.00 (0.00–0.00)	0.623	0.00 (0.00–1.54)	0.00 (0.00–1.73)	0.613
IL-21	8.15 (0.00–37.96)	10.02 (0.00–32.80)	0.995	0.00 (0.00–45.32)	0.00 (0.00–39.72)	0.709
IL-22	0.00 (0.00–92.25)	0.00 (0.00–88.29)	0.970	0.00 (0.00–44.29)	0.00 (0.00–57.38)	0.897
TNFα	0.00 (0.00–2.16)	0.00 (0.00–2.91)	0.844	2.16 (1.05–2.58)	2.58 (1.05–4.22)	0.112
**IL-17F**	—	—		**0**.**00 (0**.**00**–**2**.67)^*^	**0**.**00 (0**.**00**–**2**.06)^*^	**0**.**049**
IL-23A	0.00 (0.00–6.13)	0.00 (0.00–6.82)	0.574	0.00 (0.00–8.38)	0.00 (0.00–10.86)	0.615

^*^Serum IL-17F levels in psoriasis subjects are presented as median (range) values. A serum IL-17F level of one patient in the TC + CC group was 9.36, which was considered as an outlier. The serum levels of cytokines that were undetectable in most subjects and displayed no significant difference between the two groups, are marked with “-”; IFN-α, IL-1RA, IL-2, IL-6, IL-8, and IL-17F of control subjects.

### Clinical factors and IL17F polymorphism in psoriasis patients

Additional analyses were performed to investigate the clinical factors associated with the *IL17F* rs763780 polymorphism in psoriasis patients. There were no significant differences in the genotype distributions depending on psoriasis-related clinical parameters, including PASI score, age of onset, history of psoriatic arthritis, family history of psoriasis or two specific psoriasis phenotypes (large plaque type and guttate type) (Table 4).

**Table 4:** Analysis of clinical factors according to *IL17F* rs763780 genotypes in patients with psoriasis.

	TT group	TC + CC group	*P*-value
Number of patients	90	26	
**Age**, **years**			
Mean (SD)	39.7 (14.4)	38.7 (16.1)	0.759
**Male sex**			
Number (%)	52 (57.8)	12 (46.2)	0.294
**PASI score**			
Mean (SD)	9 (4.8)	10.1 (7.1)	0.393
**Onset age**, **years**			
Mean (SD)	28.9 (14.8)	27.4 (15.7)	0.651
**Psoriatic arthritis**			
Number (%)	4 (4.4)	1 (3.8)	0.999
**Family history**			
Number (%)	22 (24.7)	8 (30.8)	0.537
**Large plaque type**			
Number (%)	17 (18.9)	5 (19.2)	0.999
Guttate type			
Number (%)	5 (5.6)	3 (11.5)	0.376

Abbreviations: PASI, psoriasis area severity index; SD, standard deviation.

## Discussion

We observed a significant relationship between an *IL17F* polymorphism and psoriasis and found that the presence of the minor allele, C, was significantly associated with an increased risk of psoriasis development ( > 2-fold). Moreover, an increase in serum levels of cytokine, including IL-17F, was observed only in psoriasis subjects, and this increase was dependent on the presence of the polymorphism. These results suggest that the *IL17F* His161Arg polymorphism potentially affects the immune system, especially in patients with psoriasis. Sequence variations in specific nucleotides of *IL17F* can have a direct effect on the production of the encoded protein, IL-17F.

IL-12 is one of the key psoriatic cytokines in the T helper type 1 pathway^21^. In this study, a significant increase in serum IL-12 levels was observed in psoriasis subjects harbouring the *IL17F* polymorphism. It is possible that the increase in the IL-12 concentration was a secondary effect of increased IL-17F production. IL-17 and associated biomarkers activate immune cells, such as dendritic cells and macrophages, that produce IL-12^22,23^. Therefore, we suggest that the *IL17F* rs763780 polymorphism plays a critical role in the pathogenesis of psoriasis through its direct and indirect effects on the production of IL-17F and IL-12, respectively.

No significant association was evident between factors comprising manifestations of psoriasis and the *IL17F* His161Arg substitution. One possible explanation for this is that a single nucleotide mutation alone does not substantially influence disease onset, disease severity or clinical phenotypes. Further studies with more patients and investigations into additional potential contributing factors, including other significant SNPs, are required to determine the clinical effect of the His161Arg variant on psoriasis.

Very few studies on the *IL17F* rs763780 polymorphism in psoriasis have been reported to date. Our review of the literature revealed five published articles from 2015 to 2018, excluding our preliminary study. Four of these studies were conducted on European subjects (two on Spanish subjects, one on Italian subjects, and one on Polish subjects). Only one study was conducted on Asian subjects, and involved a north Indian population^24–28^. Among five studies, only one reported a relationship between the polymorphism and psoriasis susceptibility. The study conducted with the north Indian population revealed that the *IL17F* rs763780 polymorphism carried on the C allele was observed with a low frequency in psoriasis^28^, which was contrary to the results of our previous preliminary study^7^. Moreover, two of the studies reported a significant association between the polymorphism and treatment response in Spanish and Italian patients^24,26^. In the present study, as well as in our previous preliminary study, we found that the *IL17F* rs763780 polymorphism is a risk locus for psoriasis development in Asian patients^7^. With regard to the treatments administered in this study, some patients were treated with biologics: nine with ustekinumab and two with adalimumab. However, there was no significant difference in terms of treatment response according to *IL17F* genotype. Since SNPs can have effects that depend on the individual’s genetic diversity, it is important to consider race or ethnicity when classifying genes associated with psoriasis and conducting genetic studies in the future.

The *IL17F* rs763780 T/C polymorphism has been studied in various diseases other than psoriasis, and different results have been obtained depending on the disease. For example, *IL17F* rs763780 has been reported to be a susceptibility locus for Crohn’s disease^18^. Concerning ulcerative colitis, studies reported it as both a protective locus and a risk locus^19,20^. However, a meta-analysis in 2017 concluded that the polymorphism is not associated with inflammatory bowel disease^29^. With regard to asthma, a study conducted in 2006 revealed that the *IL17F* variant has a protective effect against asthma through a loss of function mechanism^10^; however, a meta-analysis conducted in 2015 reported no association with asthma^30^. A meta-analysis on rheumatoid arthritis conducted in 2017 and a meta-analysis on various cancers in 2016 reported the *IL17F* variant to be a risk locus^31,32^. The discrepancy in results obtained for different diseases suggests that the *IL17F* His161Arg mutant has a diverse spectrum of effects depending on the target organ. Based on currently available data, it is clear that *IL17F* rs763780 is an important locus for effects related to the human immune system. However, such research on psoriasis is still scarce, possibly because the *IL17F* gene has not been identified as a susceptibility locus in studies conducted on Western populations. However, the *IL17F* rs763780 polymorphism still deserves attention and is worthy of investigation based on its association with psoriasis in multi-ethnic groups.

This study has some limitations. First, it is a cross-sectional study comparing genotype and allele frequencies and serum cytokine concentrations. Hence, although the association between the polymorphism and cytokine levels was confirmed, we could not establish a causal relationship. To determine the true effect of the *IL17F* His161Arg variant on the pathogenesis of psoriasis, experimental studies and cytokine analyses using skin tissue samples are necessary. Second, we did not have complete control over factors, such as age, history of smoking, and alcohol consumption, which could affect levels of cytokines when comparing the patients and the control group. Therefore, we did not perform an analysis directly comparing cytokine levels in the control and psoriasis groups. Third, the levels of many cytokines were below the detection limit; thus, for future research, ultrasensitive assays are required. Fourth, this study was conducted in Asian patients, particularly Koreans. Large-scale studies involving additional ethnic and racial groups are needed to further confirm our results and to understand how the effects of the polymorphism vary based on ethnicity.

## Conclusion

The *IL17F* His161Arg polymorphism is a potential risk locus for psoriasis and that its variant possibly causes an increase in IL-17F and IL-12 production, leading to increased susceptibility to this condition. Further studies are required to characterise the molecular mechanisms underlying the effects of this polymorphism. Moreover, with the increasing use of biologics, prospective studies including those examining its effects on therapeutics are also required in diverse ethnic groups.

## Data Availability

The data used to support the findings of this study are available from the corresponding author upon reasonable request.
